# Reduced Antibody Responses to Vaccinations in Children Exposed to Polychlorinated Biphenyls

**DOI:** 10.1371/journal.pmed.0030311

**Published:** 2006-08-22

**Authors:** Carsten Heilmann, Philippe Grandjean, Pál Weihe, Flemming Nielsen, Esben Budtz-Jørgensen

**Affiliations:** 1 Paediatric Clinic II, National University Hospital, Rigshospitalet, Copenhagen, Denmark; 2 Institute of Public Health, University of Southern Denmark, Odense, Denmark; 3 Department of Environmental Health, Harvard School of Public Health, Boston, Massachusetts, United States of America; 4 Department of Occupational Medicine and Public Health, Tórshavn, Faroe Islands, Denmark; 5 Department of Biostatistics, Institute of Public Health, University of Copenhagen, Copenhagen, Denmark; University of Cincinnati, United States of America

## Abstract

**Background:**

Developmental exposure to polychlorinated biphenyls (PCBs) has been implicated as a possible cause of deficient immune function in children. This study was designed to assess whether prenatal and postnatal exposure to PCBs impacts on antibody response to childhood immunizations.

**Methods and Findings:**

Two birth cohorts were formed in the Faroe Islands, where exposures vary widely, because traditional diets may include whale blubber contaminated with PCBs. Prenatal exposure was determined from maternal concentrations of PCBs in pregnancy serum and milk. Following routine childhood vaccinations against tetanus and diphtheria, 119 children were examined at 18 mo and 129 children at 7 y of age, and their serum samples were analyzed for tetanus and diphtheria toxoid antibodies and for PCBs. The antibody response to diphtheria toxoid decreased at age 18 mo by 24.4% (95% confidence interval [CI], 1.63–41.9; *p* = 0.04) for each doubling of the cumulative PCB exposure at the time of examination. The diphtheria response was lower at age 7 y and was not associated with the exposure. However, the tetanus toxoid antibody response was affected mainly at age 7 y, decreasing by 16.5% (95% CI, 1.51–29.3; *p* = 0.03) for each doubling of the prenatal exposure. Structural equation analysis showed that the early postnatal exposure was the most important predictor of a decreased vaccination response.

**Conclusions:**

Increased perinatal exposure to PCBs may adversely impact on immune responses to childhood vaccinations. The clinical implications of insufficient antibody production emphasize the need for prevention of immunotoxicant exposures.

## Abstract 2

A study of two birth cohorts in the Faroe Islands, where diets may include whale blubber contaminated with polychlorinated biphenyls (PCBs), suggests exposure to PCBs may reduce immune response to childhood vaccinations.

## Editors' Summary

**Background.**

These days, mothers are as likely to worry about potential side-effects of childhood vaccinations as about whether they completely protect their child against infections. But healthy children vary in how well vaccinations “take.” After tetanus and diphtheria vaccination, for example, some children produce large quantities of antibodies that protect them against these serious bacterial diseases; others make a weaker, sometimes inadequate, immune response. What causes this variation is unclear, but one possibility is that the developing immune system is damaged in some babies by exposure both before birth and after birth through breast milk to “immunotoxicants” such as polychlorinated biphenyls (PCBs). These stable, man-made chemicals, which were widely used last century as insulators in electrical equipment and as fire retardants, accumulate and persist in the environment where they affect animal and human health. PCB-exposed babies often have a small thymus (the gland where immune system cells mature), make decreased amounts of antibodies, and have more childhood infections.

**Why Was This Study Done?**

Given these observations, could exposure to PCBs be partly responsible for the variable immunological responses of children to vaccination? If it is, and if environmental PCB levels remain high, it might be necessary to adapt more intensive vaccination programs so that all children are adequately protected against infectious diseases. In this study, the researchers examined whether prenatal and postnatal exposure to PCBs affects antibody responses to childhood vaccinations

**What Did the Researchers Do and Find?**

The researchers enrolled two groups of children—one group born in 1994–1995, the other in 1999–2001—living on the Faroe Islands in the North Atlantic. Here, people are exposed to high levels of PCBs through eating contaminated whale blubber. All the children received routine vaccinations against diphtheria and tetanus. The bacteria that cause these illnesses do so by producing a “toxoid,” so a harmless quantity of these toxic proteins is injected to stimulate a protective antibody response. For the older group, a blood sample was taken when they were seven and half years old to test for antibodies against diphtheria and tetanus toxoids; for the younger group, a sample was taken at 18 months. The exposure of the children to PCBs was assessed by measuring PCBs in their mothers' blood during pregnancy, in their mothers' milk soon after birth, and in their own blood when their antibodies were tested. The researchers found that the antibody response to diphtheria toxoid in the younger group of children was reduced by nearly a quarter for every doubling in their total exposure to PCBs. The tetanus toxoid response in the older children was reduced by a similar amount by prenatal exposure to PCBs. Although most of the children made enough antibodies to both toxoids to provide protection, about a fifth of the older children—mainly those with the highest exposures to PCBs—had worryingly low levels of diphtheria toxoid antibodies.

**What Do These Findings Mean?**

These results reveal an association between exposure to PCBs, particularly soon after birth, and a reduction in immunoprotection after childhood vaccinations. It is not clear, however, exactly how big this effect may be. This is uncertain for two reasons. First, the estimates of how much antibody responses are reduced by doubling PCB exposure are imprecise—for the younger children this change in exposure might actually have very little effect on their response to diphtheria vaccination or it could halve their response. Second, the estimates of PCB exposures are based on only three samples of body fluids so provide only a crude indication of exposure. Nevertheless, these results in children exposed to high levels of PCBs indicate that the immune function of children might also be adversely affected by the lower levels of PCBs found elsewhere in the world. Although the changes in immune function are subtle, they could be clinically important, write the researchers, and might affect both the general health of children and the degree of protection against infectious diseases that vaccination provides. Finally, these findings suggest that efforts must be stepped up to reduce PCB exposure levels to protect the sensitive immune systems of young children from these potent immunotoxicants.

**Additional Information.**

Please access these Web sites via the online version of this summary at http://dx.doi.org/10.1371/journal.pmed.0030311.

- Tox Town, a Web site available from the US National Institutes of Health, provides an introduction to toxic chemicals and environmental health risks
- US Agency for Toxic Substances and Disease Registry fact sheet on PCBs
- US Environmental Protection Agency information on PCBs
- MedlinePlus encyclopedia entries on immunization tetanus vaccine, and diphtheria vaccine
- Wikipedia pages on PCBs and vaccines (note: Wikipedia is a free online encyclopedia that anyone can edit)

## Introduction

The response of normal children to routine prophylactic vaccinations varies substantially. While reduced vaccination responses are well described with regard to specific immunodeficiency syndromes [1], the reasons for the wide variation in healthy children's production of specific antibodies are poorly understood [2]. Immunotoxic effects may be elicited by certain persistent organochlorine pollutants, such as polychlorinated biphenyls (PCBs), as indicated by decreased concentrations of immunoglobulins and increased frequencies of childhood infections in children who had been exposed to PCBs and related compounds by their mothers' contaminated diets [3–5]. Most experimental evidence [6], but not all [7], points to PCB-associated immunotoxicity being due to effects caused by dioxin-like PCB congeners.

A relevant and feasible strategy for a quantitative evaluation of the immune system of infants and small children is to measure antibody responses to immunization with thymus-dependent neoantigens [8]. Antibody formation to such antigens is dependent on antigen presentation, T lymphocyte function, and B lymphocyte function and therefore reflects overall efficacy of the immune system in relation to infection [8]. Because standardized methods for antibody assessment and extensive experience on vaccine efficacy are available, antibody responses to diphtheria toxoid and tetanus toxoid are highly suitable for this purpose [9,10].

The Faroe Islands represents a unique setting for studies of PCB immunotoxicity. While dioxin exposure is not increased, average PCB exposures are up to 10-fold higher than average levels in Northern Europe, due to the traditional habit of eating pilot whale blubber [11]. We therefore examined vaccination responses in two Faroese birth cohorts in relation to developmental immunotoxicant exposure.

## Methods

### Birth Cohorts and Clinical Examinations

Located in the North Atlantic between Shetland and Iceland, the Faroe Islands have a population of about 50,000. The present study includes prospective data from two sets of healthy Faroese mother-child pairs. The protocol was approved by the ethical review committee serving the Faroe Islands and by the institutional review board at Harvard School of Public Health.

In 1994–1995, a cohort of 182 births was established from consecutive spontaneous singleton births at term [12,13]. Prenatal PCB exposure was assessed from analyses of maternal pregnancy serum and transition milk. Major obstetric characteristics are included in Table 1. At age 7.5 y, 146 of the cohort children completed a clinical examination, and serum samples were obtained from 134 children. Five of these children were excluded, because the most recent booster vaccination had occurred less than six months before the examination. Antibody analyses were carried out on all 129 serum samples, but sufficient serum for PCB analysis was available only for 124 (group A).

**Table 1:**
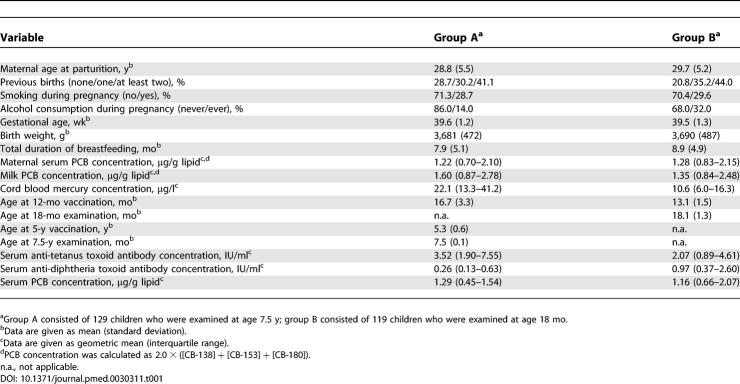
Characteristics of Two Faroese Birth Cohorts Examined at Ages 7.5 y and 18 mo

During 1999–2001, an additional birth cohort was formed using similar criteria (Table 1). A subsample (group B) of this cohort was recruited from among 279 births from a 12-mo period. Within this group, 119 children had been vaccinated in close accordance with the recommended schedule, and blood samples were taken from them at age 18 mo (sufficient serum for PCB analysis was available from 116 of these children).

Exposure levels were documented from analysis of major, persistent PCB congeners. Samples from both cohort groups included maternal serum obtained at the last antenatal examination in the 34th week of pregnancy, transition milk, and serum from the child at the time of the clinical examination (i.e., at age 54 mo and again at age 7.5 y [group A] or at 18 mo [group B]). PCB concentrations were expressed in relation to lipid concentrations [12,14]. All milk analyses were performed by the Department of Environmental Health, State Agency for Health and Occupational Safety of Schleswig-Holstein, Germany [14]. Serum analyses were conducted by the National Center for Environmental Health at the Centers for Disease Control and Prevention [15] and by the University of Southern Denmark; excellent results were obtained in intercalibration between these laboratories and in round-robins organized by the German Society of Occupational Medicine.

To avoid problems with congeners not assessed in some analyses and concentrations below the detection limit, a simplified total PCB concentration was calculated as the sum of congeners CB-138, CB-153, and CB-180 multiplied by 2.0 [11]. Because of the relevance of dioxin-like effects at increased PCB exposures, the weighted sum of the three main mono-*ortho* substituted congeners CB-105, CB-118, and CB-156 was also calculated using toxicity equivalency factors to obtain the dioxin equivalent concentration [16].

The above analyses also provided the concentrations of the pesticide metabolite *p,p*′-dichlorodiphenyldichloroethylene (*p,p*′-DDE). In addition, the mercury concentration was measured in cord blood as a measure of prenatal methylmercury exposure [12,13].

### Vaccines and Antibody Measurements

**Figure 1:**
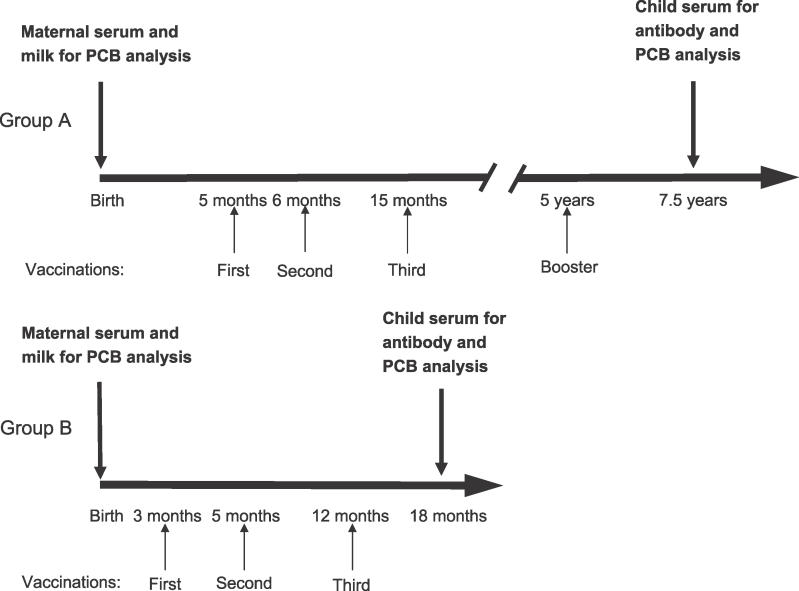
Events in Prospective Follow-Up of Two Birth Cohorts

All infants were vaccinated according to the official Danish/Faroese vaccination program, but the schedule was changed between examinations of the two cohorts (Figure 1). Tetanus toxoid and diphtheria toxoid, both classical protein antigens that depend on T helper cells for both primary and recall antibody responses [17], were included in routine childhood vaccinations at age 5, 6, and 15 months (group A), and 3, 5, 12 months (group B). Group A had later received a booster vaccination at 5 y. The immunization was performed with toxoids from Statens Serum Institut (SSI, Copenhagen, Denmark); vaccines were preserved with aluminum oxide hydrate, and none of the vaccines contained mercury-based preservatives. Children from each of the study groups A and B received the same vaccine doses. The study outcome variables were the serum-specific antibody concentrations measured by SSI using enzyme-linked immunosorbent assay calibrated with international standard antitoxins and internal standards.

### Data Analysis

Effects of PCB exposure on antibody concentrations were first determined using standard regression techniques. Antibody concentrations were log-transformed to obtain normally distributed residuals with a homogeneous variance. Models included sex, age, birth weight, maternal smoking during pregnancy, and time from last vaccination (log-transformed), as obligatory covariates. PCB exposure parameters were entered into the model, one at a time, after logarithmic transformation. Because of the logarithmic transformations, the effects could be expressed as the relative change (in percent) of the outcome variables per doubling of the exposure. Residual plots were used to assess the model fit, and regression results were also compared with the estimated dose-response functions from generalized additive models [18].

Exposure-related effects were then estimated in structural equation models with latent variables [19,20]. In these models, the observed PCB concentrations were considered manifestations of a latent true exposure, which, in turn, was assumed to affect each of the two antibody concentration outcomes. The latent exposure variable was assumed to depend on maternal blubber intake (dietary questionnaire response), and covariates were allowed to correlate with the latent exposure and the outcome. Unlike standard regression techniques, this approach takes into account measurement error in the exposure variables. In addition, a more powerful analysis is obtained, because information from different exposure markers is pooled. We first analyzed the effects of prenatal and postnatal exposures separately. Thus, all markers of prenatal exposures were considered error-prone indicators of the same latent exposure variable for exposures to PCB or the weighted mono-*ortho* congeners. All prenatal PCB exposure data were then joined into a single latent variable. A similar model was then used to determine the effect of postnatal exposures. Finally, we developed a model that allowed both these latent exposure variables to affect each outcome, with prenatal exposure at the same time as a determinant of the postnatal concentration.

We calculated the benchmark dose as the PCB exposure that increased the risk of an abnormal response from the anticipated 5%–10%. To take the statistical uncertainty into account, the lower 95% confidence limit of the benchmark dose was then calculated, in accordance with previously established procedures [21,22].

## Results

As previously observed [11,12], milk and serum concentrations of PCB congeners ranged over two orders of magnitude (Table 1) and were strongly associated with one another and also with *p,p*′-DDE (*r* = 0.88 and 0.91 for logarithmically transformed DDE and PCB in maternal serum from groups A and B, respectively). Also, the lipid-based concentrations in milk and pregnancy serum were similar. However, indicators of prenatal PCB exposure (maternal serum and milk) correlated less clearly with postnatal exposures (child serum). In addition, the correlation of PCB in maternal serum with mercury in cord blood was far from close (*r* = 0.41 and 0.32 for logarithmically transformed PCB and mercury concentrations in groups A and B, respectively).

Antibody concentrations varied widely, and the correlations between tetanus and diphtheria antibody concentrations were not close (*r* = 0.48 at age 18 mo; *r* = 0.38 at age 7 y, after logarithmic transformation). The time since the most recent vaccination influenced antibody concentrations only at age 18 mo (Table 2).

**Table 2:**
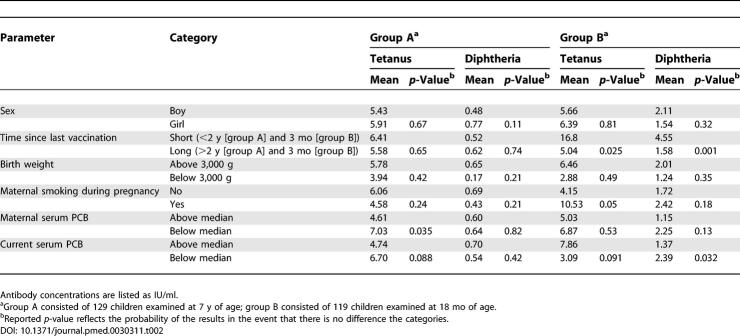
Concentrations of Serum-Specific Antibody against Tetanus and Diphtheria Toxoids in Relation to Possible Predictors

**Figure 2:**
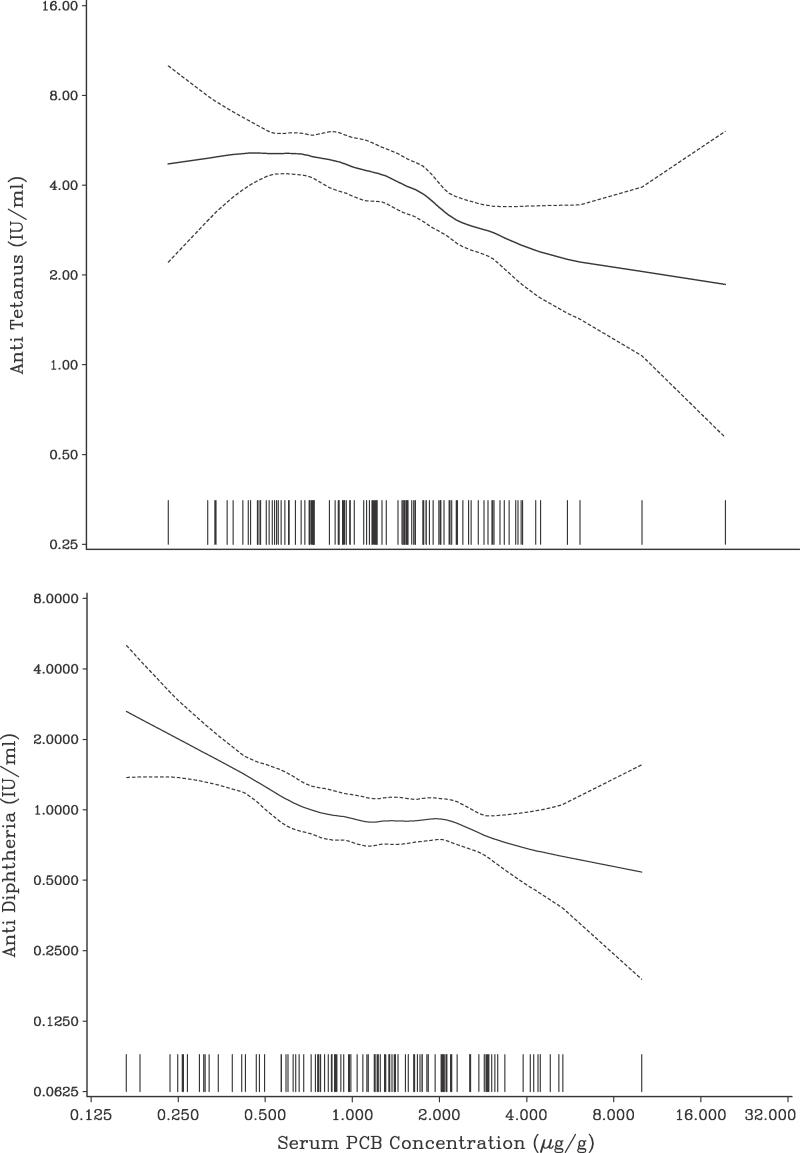
Dose-Effect Relationships between PCB Exposure and Antibody Response to Antigens from Routine Childhood Vaccinations Upper graph, maternal serum concentration of PCBs plotted against serum tetanus antibody concentrations at 7 y of age. Lower graph, maternal serum concentrations of PCBs plotted against serum diphtheria antibody concentrations, both at 18 mo of age. The broken lines indicate the 95% CIs.

At age 18 mo, the participants from group B showed negative correlation coefficients between prenatal PCB exposure and antibody concentrations, especially for the diphtheria toxoid (Figure 2; Table 3), where a doubling of the exposure resulted in a decrease by 20% in the antibody response. The PCB and the weighted mono-*ortho* PCB congeners showed similar effects on the antibody concentrations. At age 7 y group A children showed a negative effect of the prenatal PCB exposure on the tetanus toxoid antibody concentration (Figure 2; Table 3). Effects of postnatal exposures were similar. Because of the close association with PCBs that would not allow meaningful separation of the effects, *p,p*′-DDE was left out of the detailed analysis. Mercury was generally poorly associated with the antibody concentrations, with only one *p*-value below 0.3 (diphtheria in group B; *p* = 0.09). The PCB effects changed only minimally after adjustment for mercury.

**Table 3:**
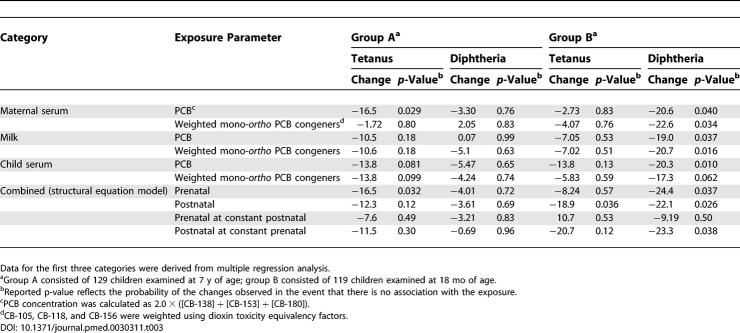
Change in Serum Antibody Concentrations after Childhood Vaccinations against Diphtheria and Tetanus Associated with a Doubling in Prenatal or Postnatal Exposure to PCBs

Most children had antibody concentrations well within the range assumed to provide protection against diphtheria and tetanus. Still, two years after the booster vaccination, 26 of the group A children (21%; 95% confidence interval [CI], 14%–28%) had diphtheria toxoid antibody concentrations below the limit for long-term protection (≥ 0.1 IU/ml).

In the structural equation model, the antibody response to diphtheria toxoid at 18 mo of age decreased by 24.4% (95% CI, 1.63%–41.9%) for a doubling of the combined prenatal PCB exposure variable (Table 3). However, the antibody concentrations were significantly affected by both prenatal and postnatal exposure. While the latter is, to a large extent, a product of the former, we found that postnatal exposure in group B was statistically significant at a constant prenatal exposure level, while the opposite was not the case. In the 7-y data from group A, the tetanus toxoid antibody concentration was primarily affected by the prenatal PCB exposure variable, with a decrease of 16.5% for each doubling of the exposure (95% CI, 1.51%–29.3%). The width of the CIs suggests that differences in PCB effects on tetanus and diphtheria, and likewise the effects of PCB and weighted mono-*ortho* PCBs, should be interpreted with caution.

For comparison with previous calculations [22], benchmark dose levels were determined for the prenatal exposure in terms of the lipid-based PCB concentration in maternal serum after covariate adjustment. The lower 95% confidence limit for the benchmark dose for the effect on the diphtheria antibody concentration in group B at 18 mo was 1.14 μg/g lipid and, for the effect on the tetanus antibody concentration in group A at 7 y, 2.18 μg/g lipid. If based on the PCB concentration in the child's serum at the time of antibody assessment, the two comparable BMDL results are 1.53 μg/g lipid and 0.84 μg/g lipid.

## Discussion

This study provides epidemiological evidence for an association between exposure to environmental pollutants and a reduction of antibody production after routine childhood immunizations. The children examined came from population-based birth cohorts and were in good health. As anticipated [9], the antibody concentrations varied substantially, while known or suspected risk factors had little impact in comparison with PCB exposure. Although statistically significant associations with PCB exposure were demonstrated, the CIs were wide (Figure 2). Results from the two different birth cohorts examined at ages 18 mo and 7.5 y showed some differences, but the tendencies seen with the tetanus and diphtheria toxoid antibody results are in overall agreement. Although the Faroese population has a substantially increased PCB exposure [23], the results of the present study suggest that possible adverse influences on the immune function may well occur also at lower exposure ranges prevalent worldwide.

The conclusions from this study must consider the uncertainty associated with the incomplete information on PCB exposure, which included analyses of body fluid samples widely separated in time. While the PCB body burden at birth reflects the mother's exposure, breast-feeding may transfer substantial amounts of the mother's accumulated PCBs via the milk fat [24]. Thus, the postnatal PCB body burden increases with the duration of the breast-feeding period [25]. The serum analyses available therefore present a crude picture of the exposure profile, and such imprecision is likely to cause an underestimation of the true effect of the exposure [26].

The Faroese population is also exposed to other seafood contaminants [12], and effects of *p,p*′-DDE and PCB could not be separated due to their close association; other potential confounders appeared to affect the results to a minimal degree. Regarding potentially causative PCB congeners, the associations may be affected by differences in their persistence. Thus, the persistent non-dioxin-like di-*ortho* PCB congeners constitute the majority of the PCB in the samples analyzed, and their concentrations will therefore tend to be fairly robust. However, the immunotoxic effects could be due to past exposures to congeners (e.g., mono-*ortho* PCBs or other dioxin-like compounds), which may no longer be present in serum in detectable concentrations, due to their shorter half-life. In this case, the more persistent PCBs may act as indicators of other congeners that caused the toxic effects. The present study cannot resolve this issue.

The group B data suggest that the antibody response may be influenced by prenatal PCB exposure, in part through its contribution to the accumulated PCB burden during the early postnatal period. The structural equation model suggested that the total perinatal exposure level was the best predictor. With regard to group A, the serum PCB concentration at age 7.5 y was still affected by prenatal and lactational transfer from the mother [15], but the current or recent PCB levels appeared less predictive of decreased antibody concentrations than those at birth.

Taken together, these findings point to the PCB burden in the early postnatal period as the major determinant of immunotoxic effects. Although this conclusion would seem plausible [2], the serum samplings were separated in time, and the exact timing of the greatest vulnerability cannot be determined from these data. In this regard, two immunological issues deserve consideration.

First, the importance of the thymus for the developing immune system is not restricted to the late gestational period, but includes early postnatal life [27]. Thus, some specific thymus-related T cell functions could likely be vulnerable to PCB-mediated toxicity during early postnatal development.

Second, the priming by the first vaccine dose before the age of 6 mo is determined by the function of the immune system at that time. The efficacy of the priming will influence the magnitude of antibody production upon later boosting. Again, this consideration would support the notion that subsequent serum antibody concentrations could be negatively affected by early postnatal PCB exposure.

Although the PCB regressions for the two vaccine toxoids were not significantly different, the negative association of PCB exposure with the diphtheria antibody response seemed more pronounced than with the tetanus antibody response at 18 mo, while the reverse was the case at age 7.5 y. In addition to statistical uncertainty, these differences need to be interpreted in light of the relative antigenicity of the toxoids, the number of boosters received, and the stability of antibody concentrations induced. Tetanus toxoid may be regarded as a stronger antigen than diphtheria toxoid and is included in the Haemophilus influenzae vaccines. The tetanus antigen stimulation was therefore more pronounced, with average antibody concentrations being somewhat higher at age 7.5 y than at 18 mo. In contrast, the mean diphtheria antibody level at age 7.5 y was only one-third of the level seen at age 18 mo, supporting the notion that this toxoid is a weaker antigen.

At the age of 18 mo (group B) all children had reached a protective antibody level after diphtheria vaccination. However, the percentage of children with low (< 0.1 IU/ml) antibody levels at age 7.5 y was surprisingly high (21%), and children with insufficient antibody concentrations had a slightly increased PCB exposure. Had the study included a comparison group with much lower PCB exposure, the high percentage of Faroese children with low diphtheria antibody concentrations could perhaps have been convincingly linked to increased PCB exposure. With regard to tetanus, the children seemed to develop high antibody concentrations at age 18 mo, but the maintenance of this high level through age 7.5 y appears sensitive to PCB immunotoxicity.

In establishing acceptable intake levels for toxic substances, regulatory agencies often calculate limits based on a benchmark dose level and an uncertainty factor. The diphtheria antibody response at 18 mo showed a lower 95% confidence limit for the benchmark dose of the same order of magnitude as the one based on PCB-related neurodevelopmental deficits [22]. With a default 10-fold uncertainty factor, the exposure limit would therefore be as low as 0.1 μg/g. Although PCB exposure levels have tended to decrease in many parts of the world [28], the present results suggest that further efforts are needed to minimize this hazard.

This epidemiological study of healthy children provides evidence of pollutant-induced reduction of antibody production following routine childhood immunizations. Although subtle, such immune function impairment could become clinically important when the immune function is challenged by other factors such as preterm birth, chronic infection, or other disease. In addition, even slight impairments could be important at a population level, e.g., with regard to an increased prevalence of respiratory tract infections.

## Abbreviations

CI: confidence interval
DDE: dichlorodiphenyldichloroethene
PCB: polychlorinated biphenyl
